# Exploring the expression and preliminary function of chicken *Gimap5* gene

**DOI:** 10.7717/peerj.7618

**Published:** 2019-09-26

**Authors:** Wanting Zhang, Sifan Xu, Guanxian Wu, Yang Liu, Qiuyuan Wang, Chaolai Man

**Affiliations:** College of Life Science and Technology, Harbin Normal University, Harbin, China

**Keywords:** chicken, *Gimap5*, Cloning, Expression, Immune response

## Abstract

GTPase immune-associated protein 5 (*Gimap5*) plays a key role in maintaining T cell homeostasis, immunological tolerance and inflammatory processes. However, there are no reports on the chicken *Gimap5* gene. In this study, the *Gimap5* gene was first cloned from chicken and characterized its tissue expression characteristics in different developmental stages. The transcriptional activities of the *Gimap5* gene in immune response were identified. The results showed that full-length cDNA sequence of* Gimap5* contained 771 bp and encoded a 256-amino acid protein. The *Gimap5* gene was transcribed in various tissues and different development stages. The transcriptional activities of *Gimap5* gene in the most tissues increased with the development of chicken, but significantly up to peak in liver and large intestine of 10-month-old chicken. The *Gimap5* gene exhibited differential transcriptional activities in immune-related tissues in immune responses, with down-regulated in liver (*P* < 0.01), spleen (*P* < 0.05) and bursa of Fabricius (*P* < 0.05), and up-regulated in thymus (*P* < 0.01). The results show that *Gimap5* may be a multifunctional gene involved in tissue function, development and immune response in chicken. These data can provide the foundation for further study of *Gimap5*.

## Introduction

*Gimap5* (GTPase of the immune-associated protein 5, also called Ian5 or Ian4) is a member of guanosine triphosphatases (GTPases) in the immune-associated protein family (MacMurray et al., 2002; Dahéron et al., 2001). Members of GIMAP family are expressed essentially in lymphocytes and hematopoietic cells (Chen et al., 2016; Chen et al., 2011). Thus far, the most conclusive results on the role of the GIMAP family members come from the studies of BB (BioBreeding) rat (Moralejo et al., 2003; Michalkiewicz et al., 2004; Diessenbacher et al., 2003). The BB rat spontaneously develops insulin-dependent diabetes and exhibits lifelong T lymphopenia (Ramanathan & Poussier, 2001; Hessner et al., 2004), and *Gimap5* was identified as the *lyp* gene in the BioBreeding diabetes-prone (BBDP) rat (Rutledge et al., 2009; Wallis et al., 2009). In BBDP rat, 215 amino acids in Gimap5 C-terminal were replaced by 19 other amino acids where transmembrane domain was truncated which leaded to lymphopenia (Hornum, Romer & Markholst, 2002). Study has shown that maintaining the quiescence of T cells requires inhibition of mTORC1 pathway activity. *Gimap5* deficiency can result in the constitutive activation of AKT/mTORC1 pathway, which causes peripheral T lymphopenia (Chen et al., 2015). *Gimap5* is associated with anti-apoptotic proteins Bcl-2 and Bcl-xL (Nitta et al., 2006). *Gimap5* can regulate thymic development and survival of T lymphocytes and take part in mitochondrial regulation of lymphocyte apoptosis by interacting with Bcl-2 family proteins (Nitta & Takahama, 2007). In addition, the *Gimap5* gene is critical for both survival and proliferation of T lymphocytes. For example, the BBDP rats can develop a spontaneous, progressive, inflammatory bowel disease. The T-lymphopenic state associated with Gimap5 deficiency rendered rats generally susceptible to T-cell-mediated autoimmunity, and defective peripheral tolerance to an intestine-specific autoantigen leaded to uncontrolled inflammation of the intestinal wall (Cousins et al., 2006). A similar situation has occurred on mice: *Gimap5*-deficiency in mice also suffered from colitis, a potential predisposing reason of which was the lymphopenic environment driving CD^4+^ T cells to acquire effector function and caused intestinal inflammation by undergoing LIP (lymphopenia-induced proliferation) (Barnes et al., 2010).

Since *Gimap5* is one of the key factors in regulating maintain T cell survival and intestinal inflammation (Cousins et al., 2006; Barnes et al., 2010; Patterson et al., 2018), it is necessary to clone and analyze *Gimap5* gene from chicken. Intestinal inflammation related diseases are one kind of the main diseases affecting poultry production. Studying chicken *Gimap5* gene may provide a positive reference for solving and improving avian enteritis-related diseases. Moreover, no reports on the *Gimap5* gene in chicken are available. In this study, the full-length coding sequence of the *Gimap5* gene in Hy-line brown chicken was isolated. The tissue transcriptional profiles of different developmental stages were analyzed. The function of the *Gimap5* gene in immune response was preliminarily studied. Our data can lay a foundation for further study of the functions and characteristics of the *Gimap5* gene.

## Material and Methods

### Ethics statement

The proposed study protocol was approved by the Institutional Animal Care and Use Committee (IACUC) of the Harbin Normal University (No. SYXKHEI2008006). All experiments in chicken were performed in accordance with the Regulations for the Administration of Affairs Concerning Experimental Animals, approved by the State Council of China.

### Animals and samples collection

16 tissues were separately collected from 14-day-old, 10-month-old, and 24-month-old Hy-line brown chickens from the Harbin Xiangfang farm, including the heart, liver, spleen, lung, kidney, brain, skeletal muscle, gizzard, thymus, skin, small intestine, large intestine, proventriculus, fat, blood, and bursa of Fabricius. Samples were snap-frozen in liquid nitrogen and stored at −80 °C.

### Cloning of *Gimap5* gene

Total RNA was extracted from 16 tissues of three different aged chickens using Trizol reagent (Invitrogen, Carlsbad, CA, USA) according to the manufacturer’s instructions, respectively. To remove genomic DNA contamination, total RNA was digested with RNase-free DNase I (Promega, Madison, WI). Using reverse transcription Kit FSQ301 (TOYOBO, Shanghai), total RNA was used to synthesize cDNA. Based on the predicted sequence of *Gimap5* from *Gallus gallus* (GenBank accession number NC_006089.5), a pair of primers were designed using the Primer Premier 5 software. Spleen cDNA was used as a template for amplifying *Gimap5* gene. The 25 µL PCR system contained 0.5 µL cDNA (20 ng/ µL), 2.0 µL dNTPs (2.5 mM, TaKaRa), 2.5 µL 10*Pyrobest buffer, 0.8 µL 10 µM forward primer (5′-ACCGACAGGCCGTGCTCCTTTGACT-3′), 0.8 µL 10uM reverse primer (5′-TGTGCCATGTGGGGACTGTGGGATT-3′), 0.1 µL high-fidelity DNA polymerase (Pyrobest, TaKaRa), and 18.3 µL sterile water. The PCR program initially started with a 94 °C denaturation for 4 min, followed by 30 cycles of 94 °C/30 s, 56 °C/30 s, 72 °C/45 s, then 72 °C extension for 10 min, finally 4 °C to terminate the reaction. PCR products were cloned into the pMD18-T vector (TaKaRa, Dalian, China) and three clones identified as positive recombinants were sequenced by BGI Tech Company. Bioinformatics analyses of the Gimap5 sequence were performed using PSIPRED, ExPaSy-prosite, SMART and Predictprotein software.

### Analysis of *Gimap5* gene expression characteristics

Quantitative reverse transcription PCR (RT-qPCR) was performed to detect the relative mRNA expression levels of the *Gimap5* gene from the different ages chicken and *β-actin* was served as an endogenous reference gene. According to the coding region of the *Gimap5* gene, qPCR primers were designed forward primer (5′-TGGTGCAGGAACGAGGGCAAGTA-3′) and reverse primer (5′-CTCTGCTTTTCATCTTCTCTCTGTA-3′). The endogenous control gene *β-actin* (GenBank: accession number NM_205518) forward primer (5′-TGGTGCAGGAGAACGAGGGCAAGTA-3′) and reverse primer (5′-CTCTGCTTTTCCTATCTTCTCTGTA-3′) were designed using Primer Premier 5 software. *Gimap5* and *β-actin* had the same qPCR dosage and reaction procedure. Each 20 µL qPCR mixture comprised 2 µL cDNA (20 ng/ µL), 0.6 µL 10 μM forward primer, 0.6 µL 10 μM reverse primer, 10 µL SYBR Green PCR master mix (TOYOBO, Shanghai), 0.4 µL ROX (TOYOBO, Shanghai) and 6.4 µL nuclease-free water. All qPCR reactions were as follows: 94 °C for 10 min, followed by 40 cycles of 95 °C 30 s, 56 °C 30 s, and 72 °C 30 s, finally 4 °C to terminate the reaction.

### Expression analysis of the *Gimap5* gene in chicken immunized with Newcastle disease vaccine

7-day-old Hy-line brown chickens were randomly divided into two groups. The chickens in the experimental group were vaccinated with LaSota (Heilongjiang Biological Production Company, Harbin, China) and chickens in the control group were treated with PBS. Five chickens were euthanized from each group on the days 14 post-vaccination (Man et al., 2015). Tissue samples were collected from the chicken, including the bursa of Fabricius, spleen, thymus, blood and liver. The expression activities of *Gimap5* gene in these tissues were analyzed by RT-qPCR (method as above).

### Statistical analysis

Three chickens were randomly selected for quantitative analysis and each sample was repeated three times. Three chickens were randomly selected for quantitative analysis and each sample was repeated three times. The relative expression activity of *Gimap5* was analyzed by qRT-PCR with 2^−Δ^Ct method (*Gimap5* vs. beta-actin). The normalized relative expression activity of *Gimap5* in immune-related tissues was analyzed by qRT-PCR with 2^−ΔΔ^Ct method ((*Gimap5* in vaccine group vs. beta-actin) vs. (*Gimap5* in control group vs. beta-actin)) (Schmittgen & Livak, 2008). One-way ANOVA of the data was obtained by SPSS 20.0 software. The data were plotted by GraphPad Prism 5 (GraphPad Software, La Jolla, CA, USA).

## Results and Discussion

**Figure 1:**
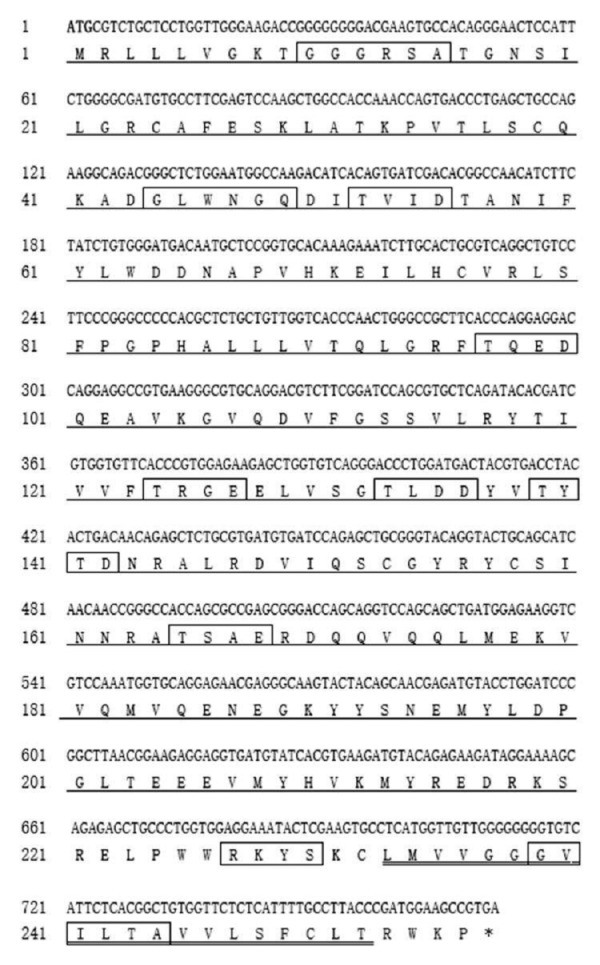
The complete CDS of Hi-line brown layer *Gimap5* gene. AIG-1 domain (1-201 AA) (single underline) is indicated; Transmembrane sequence (233-252AA) is indicated with double underline; cAMP and cGMP dependent protein kinase phosphorylation site (226-RKYS-301), Casein kinase phosphorylation sites (51-TVID-56, 96-TQED-101, 123-TRGE-128, 132-TLDD-137, 138-TYTD-143, 164-TSAE-169) , N-myristoylation sites (9-GGGRSA-16, 43-GLWNGQ-50,238-GVILTA-245) are boxed; Initiation codonsare bold text; Asterisk indicates the stop codon. The putative protein was analyzed using prospot (http://prosite.expasy.org/) SMART (http://smart.embl-heidelberg.de/) and Predictprotein (http://open.predictprotein.org) software.

Gimap5, a key regulator of lymphocyte homeostasis and hematopoietic integrity, is mainly expressed in lymphocytes and hematopoietic cells and regulates lymphocyte survival (Barnes et al., 2010). In this study, the *Gimap5* full-length coding sequence (CDS) of Hy-line brown chicken (GenBank accession number MK214431) was cloned for the first time. The sequence was consisted of 771 nucleotides and encoded 256 amino acids (AAs) (Fig. 1). The *Gimap5* gene is located on chromosome 2 and contains two exons and one intron. AIG-1 domain (1-201AA), transmembrane sequence (233-252AA) and functional sites (cAMP and cGMP-dependent protein, kinase phosphorylation site, three casein kinase phosphorylation sites and four N-my ribosylation sites) were found using the software, such as ExPaSy-prosite, SMART and Predict protein (Fig. 1). The GIMAP family proteins generally contain the AIG-1 domain and form a helix-turn-helix structure, which is an important region for DNA binding (Krücken et al., 2004).

**Figure 2:**
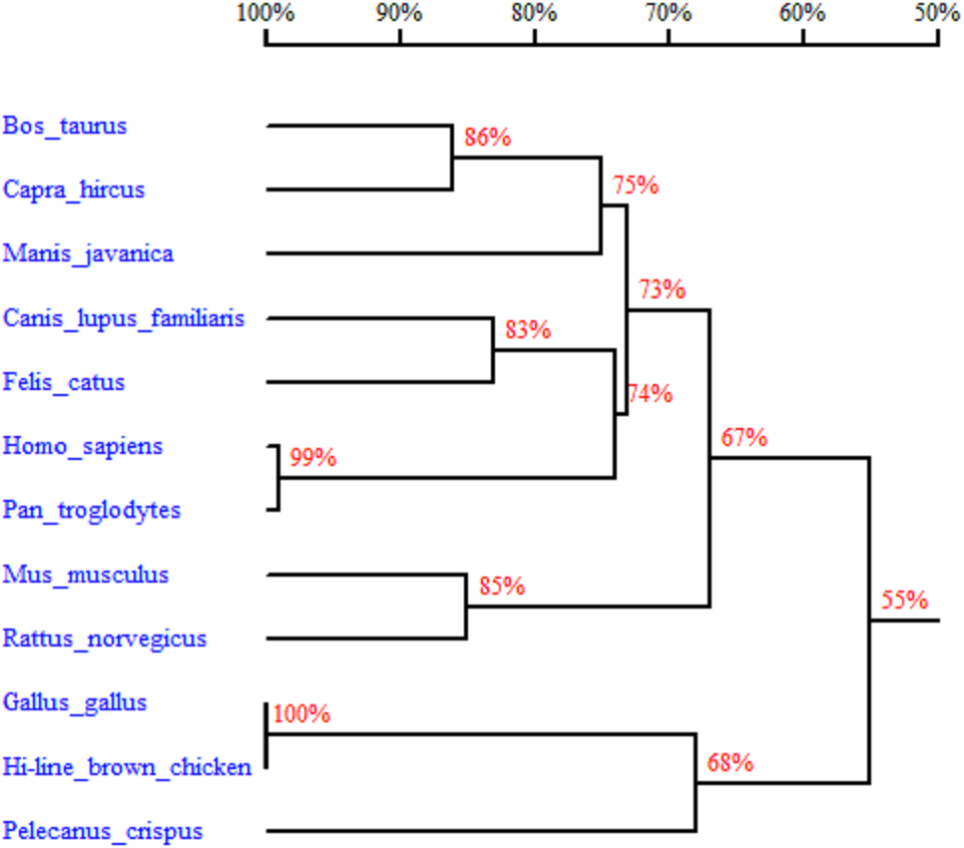
Homology tree of *Gimap5* nucleotide sequences. Analysis was done using the DNAMAN software (http://www.lynnon.com). Hi-line brown chicken (MK_214431), *Rattus norvegicus* (NM_001033913, transcript variant 1), *Homo sapiens* (NM_018384), *Mus musculus* (NM_175035), *Pan troglodytes* (XM_009454550, transcript variant 1), *Manis javanica* (XM_017651652), *Pelecanus crispus* (XM_009479064), *Bos taurus* (XM_005205795), *Canis lupus familiaris* (XM_022403865, transcript variant 1), *Gallus gallus* (XM_418519.5, transcript variant 1), *Capra hircus* (XM_018046738, transcript variant 1), *Felis catus* (XM_019825999, transcript variant 1). The percentages on the branches represent the rate of nucleotide sequence homology.

**Figure 3:**
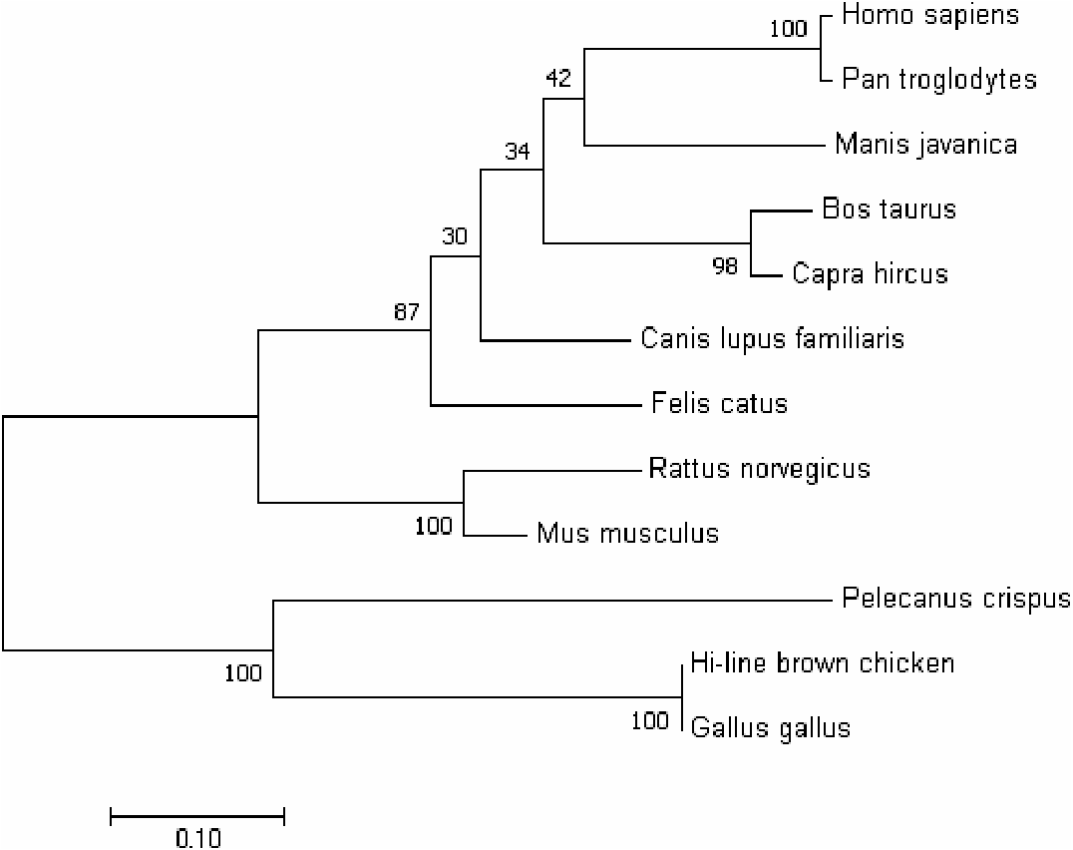
Phylogenetic tree of *GIMAP5* amino acid sequences. The phylogenetic tree was constructed using the neighbor-joining (NJ) method within MEGA 7.0. Repeat 1,000 times using Bootstrap. The number of bifurcation points represents the bootstrap value.

In order to better understand the homology and evolutionary relationship of the *Gimap5* gene with different species, *Gimap5* full-length coding nucleotide sequences from 12 species were used for homology comparison by DNAMAN software (http://www.lynnon.com). Multiple sequence alignments showed that the *Gimap5* gene was not highly conserved among different species. As a whole, *Gimap5* gene had relatively high homology within birds (over 69%) or mammals (over 67%), but the homology between the two classes was low (only 55%). Interestingly, the AA^+^ broiler *Gimap5* gene shared 100% homology with *Gallus Gallus* and only 69% homology with *Plecanus crispus* (Fig. 2). The phylogenetic tree was constructed by using MEGA7 (http://www.megasoftware.net/) on the *Gimap5* amino acid sequences of the 12 species. The result showed that the *Gimap5* amino acid sequences of mammals and birds were divided into 2 clusters. Phylogenetic tree analysis indicated that the *Gimap5* of birds was distantly related to mammals, which suggested that the *Gimap5* gene might differ from mammalian homologous genes in terms of function and characteristics (Fig. 3).

**Figure 4:**
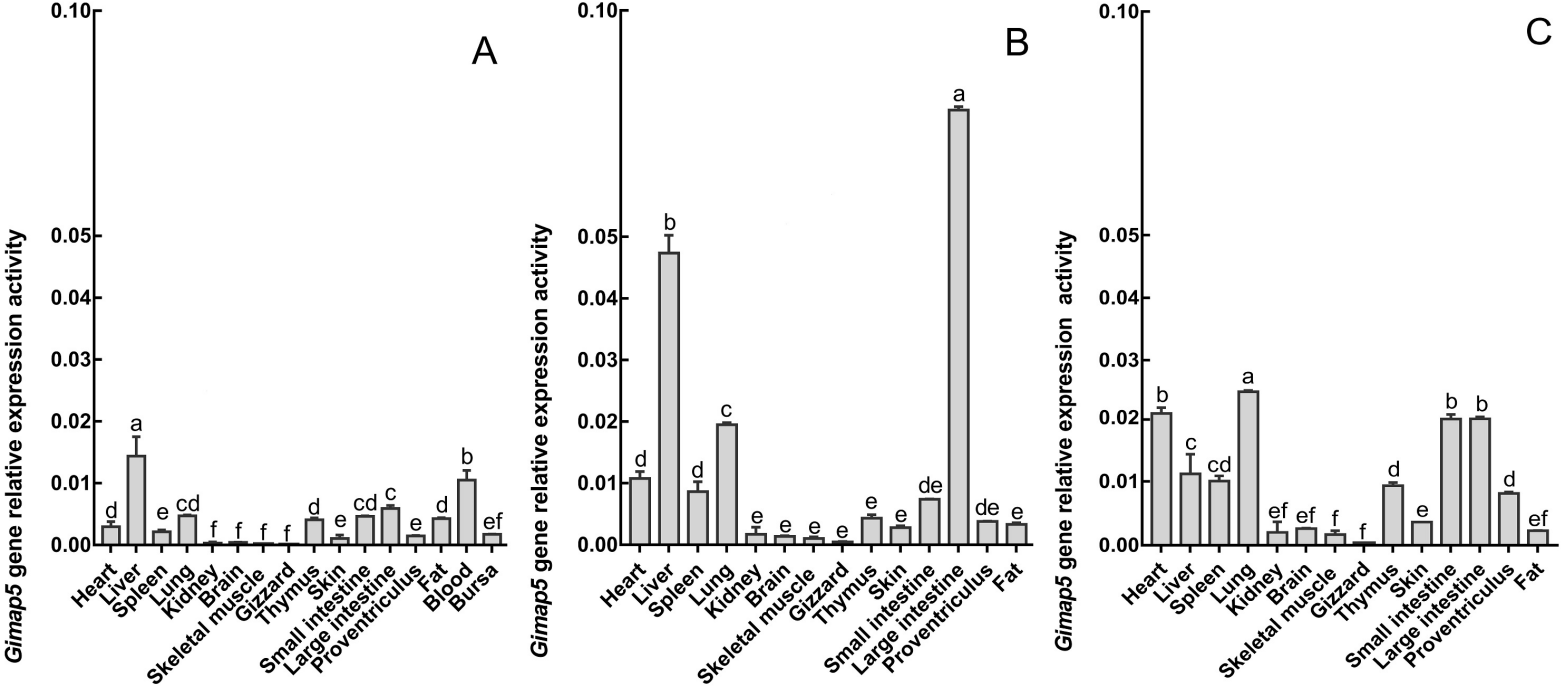
The expression pattern analysis of *Gimap5* gene in different developmental stages. (A) Expression levels of *Gimap5* in 14-day-old chicken. (B) Expression levels of *Gimap5* in 10-month-old chicken. (C) Expression levels of *Gimap5* in 24-month-old chicken. Significant difference (*P* < 0.05).

To characterize the spatiotemporal expression of chicken *Gimap5* gene, RT-qPCR was conducted to determine its transcriptional activities in different tissues of 14-day-old, 10-month-old and 24-month-old chickens, respectively. The results showed that the *Gimap5* gene was transcribed in all tissues and showed a certain regularity. According to the range of the overall transcriptional activities of the *Gimap5* gene, three levels of high, medium and low expression levels were artificially divided (Table 1). In 24-month-old chicken, the *Gimap5* gene was highly expressed (more than 0.02) in heart, lung; medium expressed (0.01–0.02) in liver, spleen, large intestine and small intestine; low expression (less than 0.01) in kidney, brain, skeletal muscle, gizzard, thymus, skin, proventriculus and fat. In 10-month-old chicken, *Gimap5* gene was highly expressed in liver and large intestine and moderately expressed in heart and lung, other tissues were low expression; In 14-day-old chicken, liver is moderately expressed, and other tissues were low expression (Fig. 4). Overall, the transcriptional activities of the *Gimap5* gene in most tissues of heart, spleen, lung, kidney, brain, skeletal muscle, thymus, skin, small intestine and proventriculus (except for liver, skeletal muscle, fat) increased with the development of chicken. It was speculated that the *Gimap5* gene might be related to the development of these tissues. It was worth mentioning that the increased transcriptional level of the *Gimap5* gene in heart, lung, small intestine and large intestine was significantly higher than other tissues. We speculated that the high expression of the *Gimap5* gene might be related to some function or development, which needs further study. Interestingly, the transcriptional activity of the *Gimap5* gene was significantly higher in the liver of 10-month-old chicken than that in other olds. Studies have shown that the *Gimap5* gene in mice was essential for maintaining normal liver function. Mice lacking *Gimap5* had a median survival of 15 weeks, exhibited chronic hepatic hematopoiesis, and in later stages showed pronounced hepatocyte apoptosis, leading to liver failure (Schulteis et al., 2008). Whether the high expression of the *Gimap5* gene in liver of 10-month-old chicken is related to liver function and development in this rapid development stage needs further study. In addition, the transcriptional activities of the *Gimap5* gene in chicken large intestine at 10-month-old was also significantly higher than other stages. Studies have shown that *Gimap5* gene mutation can impair immune lymphocyte survival and homeostasis. Gimap5** mutant mice could develop severe colitis (Barnes et al., 2010). The *Gimap5* gene was highly expressed in chicken large intestine at 10-month-old, which might be related to the intestinal development and functional characteristics (such as digestive capacity and immune status) of this stage.

**Table 1:** The transcriptional activities of the *Gimap5* gene in different developmental stages.

Olds dates tissues	14-day-old	10-month-old	24-month-old
Heart	28.0551 ± 0.0570 a	26.7953 ± 0.0522 b	26.2623 ± 0.0131 b
Liver	24.1936 ± 0.0558 b	24.7213 ± 0.0150 b	27.2931 ± 0.0011 a
Spleen	25.1576 ± 0.0391 a	24.7996 ± 0.0076 b	22.4994 ± 0.0702 c
Lung	25.6337 ± 0.0732 a	25.5134 ± 0.0098 a	25.3020 ± 0.0069 b
Kidney	30.1733 ± 0.6460 a	27.4135 ± 0.8026 c	29.4030 ± 1.0553 b
Brain	29.9128 ± 0.0698 a	29.6202 ± 0.0819 a	26.1851 ± 0.0369 b
Skeletal muscle	31.2501 ± 0.1831 a	29.6056 ± 0.3529 b	29.0718 ± 0.5532 b
Gizzard	30.0670 ± 0.0960 a	29.3493 ± 0.0444 b	28.6984 ± 0.0722 c
Thymus	26.0894 ± 0.0250 c	26.3441 ± 0.0195 a	24.8958 ± 0.0357 b
Skin	29.3877 ± 0.0245 a	27.8085 ± 0.0765 c	28.2962 ± 0.0898 b
Small intestine	24.7585 ± 0.0387 b	25.6798 ± 0.0173 a	20.7984 ± 0.0226 c
Large intestine	24.7088 ± 0.0281 b	31.8016 ± 0.0981 a	21.9052 ± 0.0395 c
Proventriculus	27.2539 ± 0.0495 a	26.2831 ± 0.0365 b	26.9190 ± 0.0442 b
Fat	27.7883 ± 0.0243 c	30.7483 ± 0.1545 a	29.6002 ± 0.1177 b

**Notes.** Letters a, b, c in table should be highlighted a,b,c: Significant differences in transcriptional activity between the same tissues of different developmental stages. Different letters represent significant differences (*P* < 0.05), while the same letters represent no significant differences (*P* > 0.05).

**Figure 5:**
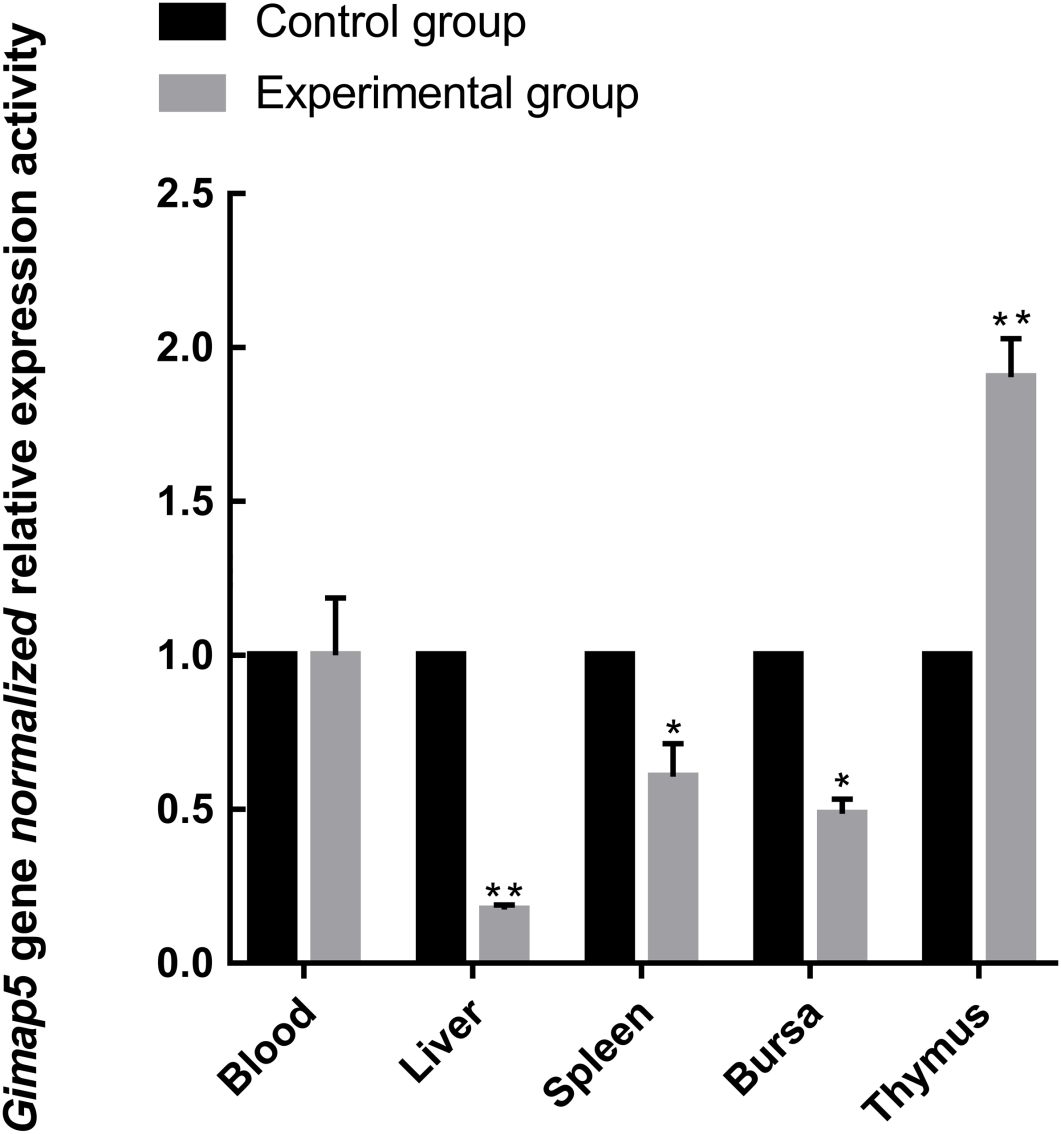
Analysis of *Gimap5* expression activity in immune response. Seven-day-old Hi-line brown chickens were vaccinated with the LaSota vaccine. The expression activity of the *Gimap5* gene from chicken 14 days post-immunization was analyzed by qRT-PCR. * or ** indicates the differences that are statistically significant at *P* < 0.05 or *P* < 0.01, respectively.

To identify the possible role of the *Gimap5* gene in chicken immune response, we examined the expression of the *Gimap5* gene in the main immune-related tissues of the chicken after vaccine immunization. Chicken administered with the LoSota vaccine expressed significantly higher hemagglutination inhibition (HI) titers against NDV in their serum, and the HI titers of the experiment group increased on day 14 post-immunization (PI) and peaked on day 21 PI (Man et al., 2015). As the linear phase of the increase in antibody titer could reflect the gene expression/activities of immune response, the immune tissues obtained from chicken 14 days PI were analyzed by RT-qPCR. The results showed that the *Gimap5* gene was down-regulated in the liver (*P* < 0.01), spleen and bursa (*P* < 0.05) of the immunized chicken, but the transcriptional activities in the thymus was significantly up-regulated (*P* < 0.01) (Fig. 5), and the *Gimap5* gene in blood did not change significantly. *Gimap5* is required for the protection of lymphocytes against cell death (Wong et al., 2010). The thymus is an important lymphoid organ, and the up-regulation of the *Gimap5* gene in the thymus may be related to the hyperplasia, differentiation and development of T lymphocytes, which might play a key role in promoting the induction of immune response in chicken thymus. Study shows that mouse Gimap5 is necessary for the survival of peripheral T cells, NK/NKT-cell development, and the maintenance of normal liver function (Schulteis et al., 2008). The *Gimap5* gene was significantly down-regulated in the peripheral immune organs (liver and spleen), which may be associated with the function of *Gimap5* in immune response. The *Gimap5* gene was down-regulated in the bursa of the immunized chicken, whether this was related to the B cell proliferation and differentiation remained to be further studied. In short, the *Gimap5* gene should be a key gene affecting the immune response.

## Conclusions

In this study, the *Gimap5* gene of Hy-line brown chicken was cloned for the first time and the spatiotemporal expression characteristics and preliminary immune function were identified. *Gimap5* was highly expressed in the liver and large intestine of 10-month-old chicken and showed significant expression changes in immune-related tissues during immune response. The study shows that *Gimap5* may be a multifunctional gene involved in tissue function, development and immune response in chicken. The results can provide direction to further study the functions and regulatory mechanisms of the *Gimap5* gene.

##  Supplemental Information

10.7717/peerj.7618/supp-1 Dataset S1 Raw data exported from the flow Real-Time PCR System applied for data analyses and preparation for Fig. 4A and Table 1

10.7717/peerj.7618/supp-2 Dataset S2 Raw data exported from the flow Real-Time PCR System applied for data analyses and preparation for Fig. 4B and Table 1

10.7717/peerj.7618/supp-3 Dataset S3 Raw data exported from the flow Real-Time PCR System applied for data analyses and preparation for Fig. 4C and Table 1

10.7717/peerj.7618/supp-4 Dataset S4 Raw data exported from the flow Real-Time PCR System applied for data analyses and preparation for Fig. 5

10.7717/peerj.7618/supp-5 Dataset S5 Raw data exported used for data analyses and Fig. 1

## Additional Information and Declarations

### DNA Deposition

The following information was supplied regarding the deposition of DNA sequences: The Gimap5 full-length coding sequence (CDS) of Hi-line brown chicken are accessible via GenBank: MK214431.

### Data Availability

The following information was supplied regarding data availability: The raw measurements are available in Datasets S1–S5.
