# Supplementary material for: Exploring the expression and preliminary function of chicken Gimap5 gene
Source: PeerJ. 2019 Sep 26;7:e7618. doi: 10.7717/peerj.7618 (PMC6766365; doi:10.7717/peerj.7618)
Supplement: Dataset S5 [file peerj-07-7618-s005.docx]

MK214431

>Seq1 [organism=Hi-Line Brown] GTPase,IMAP family member 5 (GIMAP5) gene, complete cds

ATGCGTCTGCTCCTGGTTGGGAAGACCGGGGGGGGACGAAGTGCCACAGGGAACTCCATTCTGGGGCGATGTGCCTTCGAGTCCAAGCTGGCCACCAAACCAGTGACCCTGAGCTGCCAGAAGGCAGACGGGCTCTGGAATGGCCAAGACATCACAGTGATCGACACGGCCAACATCTTCTATCTGTGGGATGACAATGCTCCGGTGCACAAAGAAATCTTGCACTGCGTCAGGCTGTCCTTCCCGGGCCCCCACGCTCTGCTGTTGGTCACCCAACTGGGCCGCTTCACCCAGGAGGACCAGGAGGCCGTGAAGGGCGTGCAGGACGTCTTCGGATCCAGCGTGCTCAGATACACGATCGTGGTGTTCACCCGTGGAGAAGAGCTGGTGTCAGGGACCCTGGATGACTACGTGACCTACACTGACAACAGAGCTCTGCGTGATGTGATCCAGAGCTGCGGGTACAGGTACTGCAGCATCAACAACCGGGCCACCAGCGCCGAGCGGGACCAGCAGGTCCAGCAGCTGATGGAGAAGGTCGTCCAAATGGTGCAGGAGAACGAGGGCAAGTACTACAGCAACGAGATGTACCTGGATCCCGGCTTAACGGAAGAGAAGGTGATGTATCACGTGAAGATGTACAGAGAAGATAGGAAAAGCAGAGAGCTGCCCTGGTGGAGGAAATACTCGAAGTGCCTCATGGTTGTTGGGGGGGGTGTCATTCTCACGGCTGTGGTTCTCTCATTTTGCCTTACCCGATGGAAGCCGTGA
